# Presence of sandfly-borne phleboviruses of two antigenic complexes (*Sandfly fever Naples virus* and *Sandfly fever Sicilian virus*) in two different bio-geographical regions of Tunisia demonstrated by a microneutralisation-based seroprevalence study in dogs

**DOI:** 10.1186/s13071-014-0476-8

**Published:** 2014-10-12

**Authors:** Sonia Sakhria, Sulaf Alwassouf, Wasfi Fares, Laurence Bichaud, Khalil Dachraoui, Cigdem Alkan, Ziad Zoghlami, Xavier de Lamballerie, Elyes Zhioua, Remi N Charrel

**Affiliations:** Institut Pasteur de Tunis, Laboratory of Vector Ecology, Tunis, Tunisia; Aix Marseille Université, IRD French Institute of Research for Development, EHESP French School of Public Health, EPV UMR_D 190 “Emergence des Pathologies Virales”, 13385 Marseille, France; IHU Méditerranée Infection, APHM Public Hospitals of Marseille, 13385 Marseille, France

**Keywords:** Phleboviruses, Toscana virus, Sand flies, Dogs, Sentinels, Emerging, Mediterranean basin, Bunyaviridae

## Abstract

**Background:**

Sandfly-borne phleboviruses are present in North Africa where they can infect humans in regions where *Leishmania infantum,* the causative agent of zoonotic visceral leishmaniasis in the Western Mediterranean basin is present affecting both humans and dogs. We investigated the capacity of dogs to be used as sentinels for sandfly-borne phleboviruses as previously shown for leishmaniasis.

**Findings:**

A total of 312 sera were collected from guard dogs in two different bioclimatic regions (governorates of Kairouan and Bizerte) of Tunisia where zoonotic visceral leishmaniasis has been reported. These sera were tested for the presence of neutralising antibodies against 3 phleboviruses: Toscana virus, Punique virus and Sicilian virus. In the governorate of Kairouan, seroprevalence rates of 7.5%, 43.5%, and 38.1% were observed for Toscana, Punique and Sicilian virus, respectively. A high proportion of sera from the governorate of Bizerte were hemolyzed and showed high cytotoxicity for the cells and subsequently precluded detailed interpretation of this batch. However, validated results for 27 sera were in agreement with data observed in the governorate of Kairouan.

**Conclusions:**

Toscana virus is present in the governorate of Kairouan but at a lower rate compared to Punique and Sicilian viruses. These three sandfly-borne phleboviruses can infect dogs. Direct detection and isolation of the viruses are now to be attempted in animals as well as in humans. Our findings showed that guard dogs are good sentinels for virus transmitted by sandflies and strongly suggested that the high seroprevalence rates observed in dogs merit further attention.

## Background

Recent studies have indicated that sandfly-borne phleboviruses (genus *Phlebovirus*, family *Bunyaviridae*) were not only geographically restricted to southern Europe, but were also present in North Africa [1-6]. At least, viruses of 3 different antigenic complexes are transmitted by sandflies in the Old World: Salehabad complex, Sandfly fever Sicilian complex and Sandfly fever Naples complex. Two phleboviruses of the Sandfly fever Naples complex were reported to be present in Tunisia, namely Toscana virus and Punique virus as assessed by virus isolation [2,7]. Another virus, provisionally named Utique virus that belongs to the Sandfly fever Sicilian complex was genetically detected in Tunisia but it has not been isolated yet [7]. A recent sero-epidemiological study conducted in the governorate of Bizerte (northern Tunisia) showed that both Toscana and Punique viruses could infect human populations although Toscana virus was much more prevalent than Punique virus [5]. In Tunisia, Toscana, Punique and Utique viruses were detected and/or isolated from *Phlebotomus pernicious* and *Phlebotomus longicuspis,* which are considered as possible vectors of phleboviruses [2,7]. Both sandfly species are also the main vectors of *Leishmania infantum*, etiologic agent of zoonotic visceral leishmaniasis (ZVL) in Tunisia [8-10].

Dogs are the main reservoir host of *L. infantum* and therefore they are used as sentinels to assess the risk of ZVL and other zoonotic vector-borne diseases [11]. *Phlebotomus pernicious* and *P. longicuspis* are widely distributed in Tunisia [12], and subsequently, we hypothesized a large distribution of sandfly-borne phleboviruses.

It is important to point out that the governorate of Kairouan is the most endemic foci for ZVL [10]; in addition approximately 40% of the rural human population living in the governorate of Bizerte possesses antibodies neutralizing Toscana virus (TOSV-NT-Ab) [5]. Therefore, human populations are exposed to sandfly-borne diseases in both governorates.

## Findings

**Figure 1:**
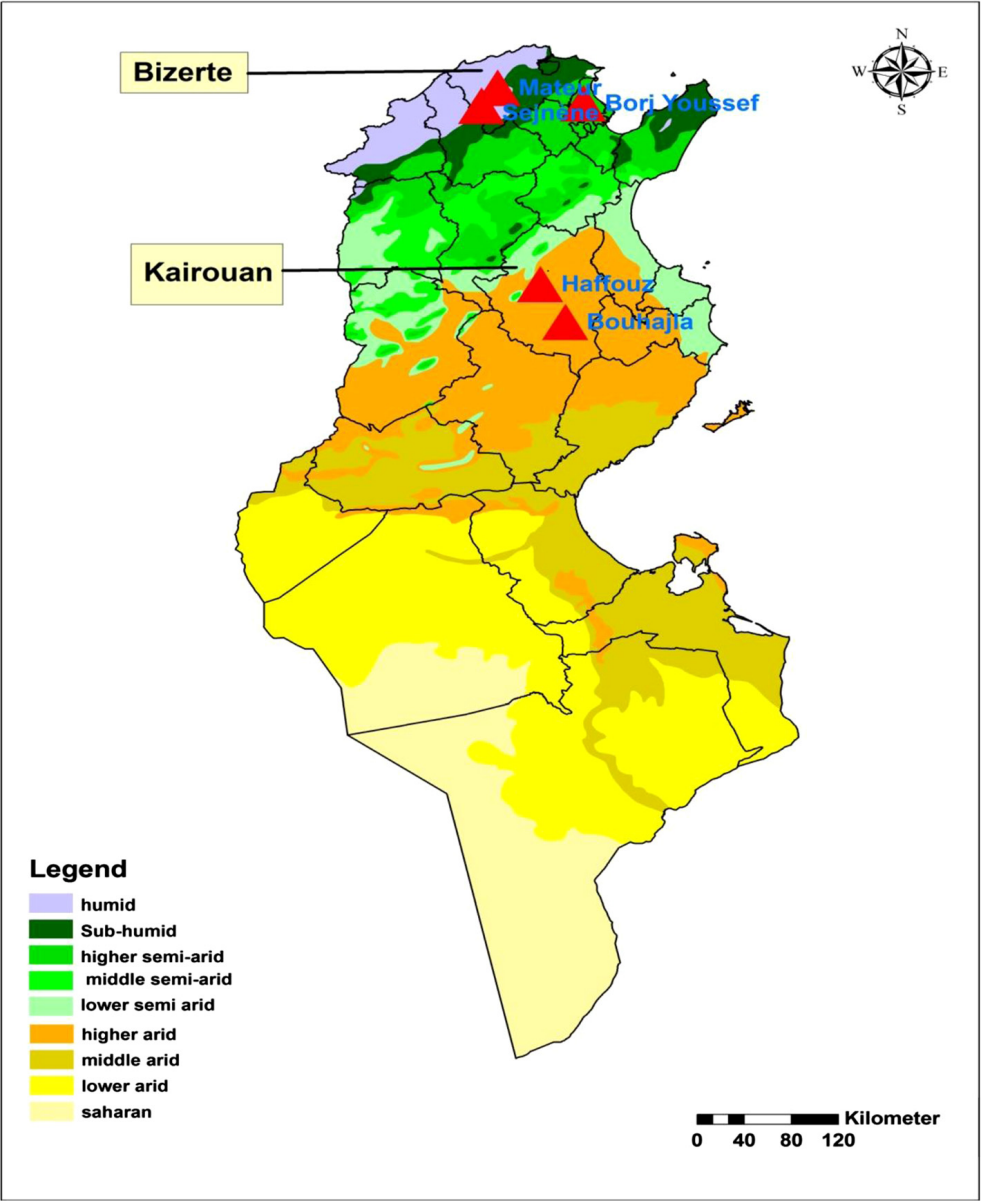
**Sampling sites of sera from dogs.**

The study took place in the governorate of Bizerte located in Northern Tunisia and in the governorate of Kairouan located in Central Tunisia corresponding to two different bio-geographical areas (Figure 1). To assess the circulation of sandfly-borne phleboviruses, dogs were used as sentinel. A retrospective study on dogs was undertaken in several districts of the governorates of Bizerte and Kairouan during the fall of 2013. Sampling was performed in five locations belonging to different bio-climatic zones varying from humid to arid (Sejnane: 36°56’ N, 9°21’ E, humid; Mateur: 37° 03’ N, 9° 28’E, Sub-humid; Borj Youssef; 36°56’N, 10°07’E, semi-arid; Haffouz: 34°51 N, 9°29’E, arid; Bouhajla, 35°24’N, 9°56’ E, arid) (Figure 1). The selected sites were restricted to previously surveyed areas characterized by the abundance of phlebotomine species of the subgenus *Larroussius* [2,10,12]. The typical setting of a house in endemic areas for ZVL includes the guard dog, attached for his entire life close to the house, and to the sheep shed and chicken henhouse. Houses are always surrounded by cactus to provide protection against trespassing and cactus peers are highly appreciated by villagers. This ecological setting offers suitable biotope for sandflies. Sheep sheds made usually with mud walls are breeding sites for sandflies (Zhioua, unpublished data). Flowers of the cactus are the only sugar source available around. Animals located in the peridomestic areas are the main source of blood meal including humans for sandflies. Dogs are the main source of *L. infantum* infection to sandflies. We organized door-to-door visits with a local veterinarian and a health worker who introduced the team to the local population. Information regarding age, sex, race was obtained after interviewing dog owners who gave their consent to be involved in the study. After filling out the questionnaire, each dog was examined clinically by the veterinarian and a 2-ml blood sample was collected by venepuncture of the forelimb. This study was performed following approval from the IACUC of Pasteur Institute of Tunis, Tunisia IPT/UESV/19/2010.

Dog sera were tested by using a microneutralisation assay performed comparatively with (i) two viruses belonging to the Sandfly fever Naples species (Toscana virus and Punique virus), (ii) and one virus belonging to the Sandfly fever Sicilian species (Sicilian virus) as described previously [5]. Neutralisation is the most discriminative serological assay that is well-adapted to differentiate the affinity of antibodies against different viruses. In addition, there is almost no cross-reaction.

From the governorates of Kairouan and Bizerte, 194 and 118 sera were collected from guard dogs, respectively. The virus microneutralisation (VNT) assay was performed in 96-well microtitre plates using Vero cells as previously described [5] with slight technical modifications. Briefly, two-fold serial dilutions from 1:10 to 1:80 of a 50 μL-serum was mixed with an equal volume of virus culture titered at 1000 TCID50 into 96-well plates, providing two-fold final dilutions from 1:20 to 1:160.

For this study the 3 virus strains were (i) Toscana virus strain MRS2010-4319501 [13], (ii) Punique virus strain Tunisia2009T101 [7], (iii) Sandfly fever Sicilian virus strain Sabin [14]. The plate was incubated at 37°C for one hour, then a 100 μL volume of Vero cells containing approximately 2.10^4^ cells in 5% foetal bovine serum was added to each well, and incubated at 37°C in presence of 5% CO_2_. After 5 days, the microplates were read under an inverted microscope, and the presence or absence of cytopathic effect was noted. The titre (no neutralisation, neutralisation at 1:20, 1:40, 1:80 and 1:160) was recorded. The threshold for positivity was defined as 1:20 [5].

The results are presented in Table 1. Of the 194 sera originating from Kairouan, 47 were cytotoxic for the cells, and therefore calculations were done on the basis of 147 sera. In the governorate of Bizerte, only 27 sera were considered as suitable for calculation because the proportion of sera showing cytotoxicity was much higher. Thus, the very low number precluded any statistical calculations. Cytotoxicity was frequently observed with hemolyzed sera, resulting from suboptimal conditions of storage.

**Table 1:** **Microneutralisation-based seroprevalence rates for Toscana, Punique and Sicilian viruses on 312 sera collected from guard dogs in Tunisia**

**Tested sera**	**Cytotoxic for Vero cells**	**Toscana virus n (%)**	**Punique virus n (%)**	**Sicilian virus n (%)**
**Kairouan, n = 194**	47	11 (7.5%)	64 (43.5%)	56 (38.1%)
**Distribution of VNT titres neg/20/40/80/160**		136/6/5/0/0	83/2/8/11/43	91/4/24/23/5
**Bizerte, n = 118**	91	0	2 (7.4%)	16 (59.2%)
**Distribution of VNT titres neg/20/40/80/160**		0	25/0/0/1/1	11/0/10/6/0

The results obtained from the analysis of 147 sera from Kairouan showed that the 3 viruses (or very closely related viruses) were circulating in the region. Seroprevalence rates of 43.5%, and 38.1% were observed for Punique virus and Sicilian virus, respectively. In contrast, only 7.5% of dog sera possessed TOSV-NT-Ab. Of the 11 sera that showed positive results with Toscana virus, 7 did not contain PUNV-NT-Ab demonstrating that these dogs had been infected with Toscana virus only and not with Punique virus. For the 4 remaining sera, the respective titres for TOSV-NT-Ab/PUNV-NT-Ab were 40/40 for one serum, 40/160 for one serum, and 20/160 for 2 sera. Three sera showed a difference ≥ two-fold dilutions in favour of Toscana virus, which is undisputable evidence of Toscana virus past infection. Therefore, past infection with Toscana virus is unambiguous for 10 of the 11 reactive sera. Since neutralisation is the most specific and discriminative serological technique; we can exclude that cross-reactivity is a valid explanation for the finding of NT Ab against two related viruses. Thus, it is most likely that these dogs have been successively infected by Toscana virus and Punique virus. Toscana virus is present in the governorate of Kairouan although it is much less frequently infecting dogs than Punique and Sicilian viruses.

In the governorate of Kairouan, the presence for TOSV-NT-Ab in only 7% of dogs versus 43% of PUNV-NT-Ab was unexpected; indeed, it is in contrast with the high rate of TOSV-NT-Ab and low rate of PUNV-NT-Ab recently reported in humans from the governorate of Bizerte [5]. A possible explanation lies in differences of the phlebotomine fauna present in the two governorates. While *P. perniciosus* is the predominant sandfly species in the governorate of Bizerte, *P. longicuspis* is the most abundant sandfly species in the governorate of Kairouan [2,10,12]. Since both sandfly species are shown to be vector of phleboviruses [7], it is well conceivable that *P. perniciosus* is more anthropophilic compared to *P. longicuspis* which is more zoophilic leading to a difference in human versus dog biting rates and subsequently to a difference in the infection status with Toscana virus and Punique virus in humans compared to dogs within the governorates of Bizerte and Kairouan. To address this hypothesis, the forage ration of the two sandfly species needs to be determined in both governorates.

The high rate of SFSV-NT-Ab observed in the governorate of Kairouan demonstrates that Sicilian virus or a very closely virus related to Sicilian virus is present and circulates at high levels in the region. Such findings are in agreement with the identification of sequences corresponding to a Sicilian-like virus, provisionally named Utique virus in the village of El-Felta located within the governorate of Sidi Bouzid adjacent to the governorate of Kairouan [7].

The results obtained from the 27 sera collected from dogs in the governorate of Bizerte for which the VNT was interpretable showed that 16 sera contained neutralising antibodies against Sicilian virus which is coherent with the detection of Utique virus in 7 pools of sandflies collected from the same region in 2010 [7]. The results observed with Toscana virus (absence of positive serum), and Punique virus (n = 2; 7.4%) cannot be extrapolated because of the low numbers, but confirms that Punique virus can infect not only humans [5] but also dogs.

The results of this study provided further evidence that Toscana, Punique and Sicilian viruses are present in Tunisia and showed that guard dogs may represent excellent sentinels for virus transmitted by sandflies. The existence and nature of vertebrate reservoir of sandfly-borne phleboviruses is unknown; however, the high seroprevalence rates observed in dogs in this study leads to further investigations concerning the possible role of dogs in the transmission dynamic of theses arboviruses.

Whether Punique virus and Sicilian virus represent a threat for humans in Tunisia, as previously shown for Toscana virus [5], needs to be addressed in the future. For this, entomological studies combined with virological investigation should be organized as well as clinical studies in regional hospitals.

## Conclusions

In conclusion, the results of this study showed that Toscana, Punique and Sicilian viruses are circulating in several regions of Tunisia, and dogs are frequently infected with these viruses for which they could serve as sentinels.
